# Overview of the Diversity, Phylogeny and Biogeography of Strombidiid Oligotrich Ciliates (Protista, Ciliophora), With a Brief Revision and a Key to the Known Genera

**DOI:** 10.3389/fmicb.2021.700940

**Published:** 2021-09-16

**Authors:** Wen Song, Dapeng Xu, Xiao Chen, Alan Warren, Mann Kyoon Shin, Weibo Song, Lifang Li

**Affiliations:** ^1^Laboratory of Marine Protozoan Biodiversity and Evolution, Marine College, Shandong University, Weihai, China; ^2^State Key Laboratory of Marine Environmental Science, College of Ocean and Earth Sciences, Xiamen University, Xiamen, China; ^3^Department of Life Sciences, Natural History Museum, London, United Kingdom; ^4^Department of Biological Science, University of Ulsan, Ulsan, South Korea; ^5^Laboratory for Marine Biology and Biotechnology, Qingdao National Laboratory for Marine Science and Technology, Qingdao, China

**Keywords:** ciliary pattern, identification, morphology, Oligotrichia, planktonic ciliates

## Abstract

Strombidiids are common free-living ciliates that have colonized coastal and open oceanic waters across the world. In recent years, numerous new taxa and gene sequences of strombidiids have been reported, revealing a large diversity of both their morphologic and genetic features. Here, we compare the taxonomic characters of all genera in the family Strombidiidae, provide a key to their identification, and investigate their molecular phylogeny. In addition, we analyze their regional distribution based on faunal data accumulated in China and attempt to infer their global distribution based on SSU rRNA gene sequence data. The current work revises the systematics of strombidiids based on morphologic, phylogenetic, and biogeographic evidence and provides a genus-level review of marine strombidiids.

## Introduction

Since the proposal of the “microbial loop” hypothesis (Azam et al., 1983), ciliates have been recognized as one of the major components of marine ecosystems (Azam and Malfatti, 2007; Fenchel, 2008; de Vargas et al., 2015; Bai et al., 2020a,b; Wang R. et al., 2020; Liu Q. et al., 2021). Ciliates, especially species in the subclass Oligotrichia, play essential roles in the microbial loop as grazers of nano-/picoplankton and as food sources for larger zooplankton including copepods and fish larvae (Song et al., 2009; Hu et al., 2019; Huang et al., 2021). Consequently, oligotrichs have been a focus in the study of marine science, especially their diversity, phylogeny and biogeography (Song et al., 2009; Santoferrara and McManus, 2017; Hu et al., 2019).

Members of the family Strombidiidae Fauré-Fremiet, 1970, are reported to be ubiquitous and to episodically dominate the microzooplankton community in both coastal and oceanic waters (Sherr and Sherr, 1987; Dolan et al., 2013; Sun et al., 2019; Yang et al., 2020). Due to their wide distribution, high abundance, and fast growth rate (Montagnes, 1996; Santoferrara and McManus, 2017), numerous studies focusing on the taxonomy and phylogeny of strombidiids have been carried out (e.g., McManus et al., 2010; Agatha, 2011, 2014; Lee et al., 2015; Liu et al., 2015a, 2016; Liu W. et al., 2017; Gao et al., 2016, 2017; Li et al., 2020). The somatic ciliature of most strombidiids usually includes a girdle kinety (GK) and a ventral kinety (VK) although there is considerable variation of these two among the 12 genera of the family Strombidiidae and in some species the ventral kinety is lacking (Lynn, 2008; Song et al., 2009; Agatha, 2011). Since the first strombidiid species, *Strombidium sulcatum* (Claparède and Lachmann, 1859), was described, over 100 nominal species have been reported worldwide (Shen et al., 2018; Wang et al., 2018, 2019; Küppers et al., 2019; Song et al., 2019; Liu W. et al., 2021). Nevertheless, although the systematics of strombidiids have been frequently discussed, there are many disparities between the molecular and morphological data, especially at the genus and species levels (McManus and Katz, 2009; Agatha and Strüder-Kypke, 2014).

Since strombidiids are common members of the microzooplankton community, their distribution and biogeography have received extensive attention from ecologists. Knowledge of this topic, however, remains scant because data on their biogeography are limited by undersampling worldwide (Agatha, 2011; Lu et al., 2020). In recent years, the diversity of strombidiids in Chinese coastal waters has been extensively studied and almost all known genera in the family Strombidiidae have been recorded (Song W. et al., 2013; Liu W. et al., 2017; Song et al., 2018, 2019; Liu et al., 2019; Hu et al., 2019). Consequently, there is sufficient available data for their distribution in coastal regions of China to be reviewed.

In the last two decades, environmental sequencing-based techniques, e.g., clone library construction, denaturing gradient gel electrophoresis (DGGE), restriction fragment length polymorphism (RFLP), and high-throughput sequencing (HTS), have enabled studies on the distribution of ciliates to be carried out in a culture-independent and taxonomic expertise-free way (López-García et al., 2001; Moon-van der Staay et al., 2001; de Vargas et al., 2015; Xu et al., 2017). The application of environmental sequencing-based techniques has prompted the discovery of new ciliate lineages and previously undiscovered patterns of distribution (Grattepanche et al., 2016; Santoferrara et al., 2016; Sun et al., 2016, 2019; Wang Y.R. et al., 2020). The rapid growth in the number of available SSU rRNA gene sequences allied to detailed observation and documentation of morphological characteristics can thus serve as a reliable database for inferring the taxonomic assignments of environmental sequences. The vast amount of data accumulated in the GenBank database, especially the nearly full-length SSU rRNA gene sequences from clone library studies, also offer the opportunity to infer the biogeographical distribution of strombidiids.

The aims of the present study are to (1) review the systematics of all genera in the family Strombidiidae based on morphological and, where available, molecular data; (2) provide a key to the identification of these genera, and (3) investigate the distribution of marine strombidiids based on taxonomic and SSU rRNA gene sequence data.

## Materials and Methods

### Phylogenetic Analyses

The SSU rRNA gene sequences of 65 species of Oligotrichia and Choreotrichia obtained from GenBank were employed to construct phylogenetic trees. Five species (representing five genera) of Halteriida and Hypotrichia were used as outgroup taxa. All 70 sequences were aligned using the MUSCLE algorithm on the GUIDANCE web server with the default parameters (Penn et al., 2010a, b). The ends of the alignment were trimmed using Bioedit (Hall, 1999), yielding a matrix of 1,768 characters. Maximum likelihood (ML) analysis was conducted using RAxML-HPC2 on XSEDE v 8.2.12 (Stamatakis, 2006; Stamatakis et al., 2008) with the optimal model evaluated by the online server CIPRES Science Gateway (Miller et al., 2010). The reliability of internal branches was assessed with a non-parametric bootstrap method featuring 1,000 replicates.

Bayesian inference (BI) analysis was performed with MrBayes 3.2.6 on XSEDE v 3.2.6 (Ronquist and Huelsenbeck, 2003) provided on the CIPRES Science Gateway, with the model GTR+I+Γ selected by the Akaike information criterion (AIC) in MrModeltest v2 (Nylander, 2004). Markov chain Monte Carlo chains were run for 4 × 10^6^ generations with two parallel runs, each with four simultaneous chains, sampling every 100 generations. The first 10,000 generations were discarded as burn-in prior to construction of the consensus tree.

### Biogeographic Distribution

**FIGURE 1:**
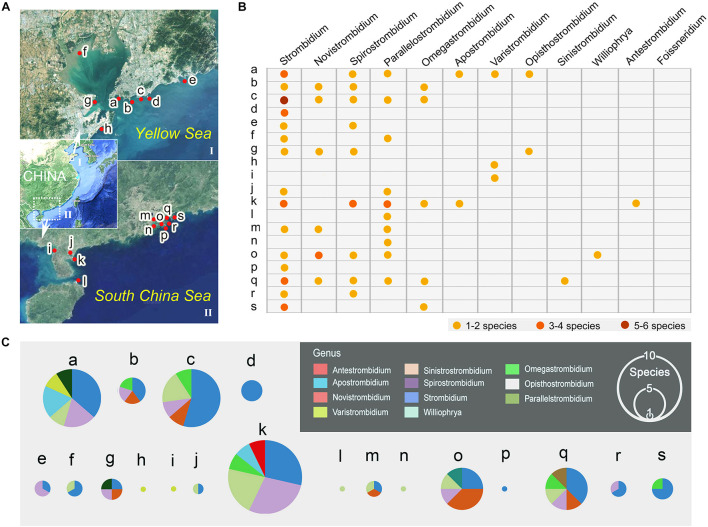
Geographic distribution of the 12 genera of the family Strombidiidae in China. **(A)** Showing sampling sites in northern (I) Chinese coastal waters near Qingdao, Shandong Province, and in southern (II) Chinese coastal waters in Guangdong Province and Hainan Province. Red spots on the map mark the locations where species were collected. Location a: Intertidal zone in Qingdao. b: Intertidal zone in Qingdao. c: Coastal waters in Qingdao. d: Beach and reef in Qingdao. e: Beach and reef in Qingdao. f: Intertidal zone in Jiaozhou Bay. g. Intertidal zone in Huangdao. h. Beach in Huangdao. i: Intertidal zone in Zhanjiang. j: Coastal waters in Zhanjiang. k: Mangrove wetland in Zhanjiang. l. Estuary in Haikou. m. Estuary in Guangzhou. n. Coastal waters in Zhuhai. o. Mangrove wetland in Shenzhen. p. Dock in Shenzhen. q. Intertidal zone in Huizhou. r. Mariculture zone in Huizhou. s. Mangrove wetland in Daya Bay. **(B)** The table shows the occurrence and species diversity of each genus at each sampling site. **(C)** The pie charts show the species composition of each sampling site, the size of the pie indicates the species richness in each site.

The regional biogeographic patterns of strombidiids were inferred based on a compilation of faunal data from samples collected from coastal regions of China. Variations in the distribution of strombidiid species from 19 coastal sites (Figure 1) were analyzed. The species richness and the community composition in each site were compared. In addition, among these sites, eight (a–h in Figure 1) were located near Qingdao, Shandong Province, northern China, and 11 (i–s in Figure 1) were located in Guangdong and Hainan Provinces, southern China. The communities were compared between northern and southern China.

To infer the possible worldwide biogeographic distribution of the strombidiids, each annotated SSU rRNA gene sequence was blasted against the GenBank The nucleotide collection database and it’s nearest environmental neighbor (NEN), i.e., the environmental sequence with the highest sequence similarity, were retrieved (Xu et al., 2020). The locations of the sequences of identified strombidiids and NEN retrieved sequences were marked on a map.

## Results and Discussion

To date, more than 100 species belonging to 12 genera have been assigned to the family Strombidiidae, including 10 species and seven genera that have been described during the past decade (Agatha, 2011; Liu W. et al., 2017; Hu et al., 2019). In addition, molecular data on strombidiids have been extensively collected in the past 15 years and there are now 46 species with available SSU rRNA gene sequences (Gao et al., 2017; Song et al., 2020). Although evolutionary relationships of strombidiids have been discussed in several studies, the molecular phylogenies often reveal unexpected relationships that are not consistent with cladograms based on morphological data, especially at the genus level (McManus and Katz, 2009; Agatha and Strüder-Kypke, 2014). The number of new species and genera in the family Strombidiidae is continually increasing, supporting the assertion that 83%–89% of free-living ciliate species are undescribed (Foissner et al., 2008). The taxonomy and systematics of the family Strombidiidae therefore need to be updated.

### Taxonomic Review of Genera in the Family Strombidiidae

**FIGURE 2:**
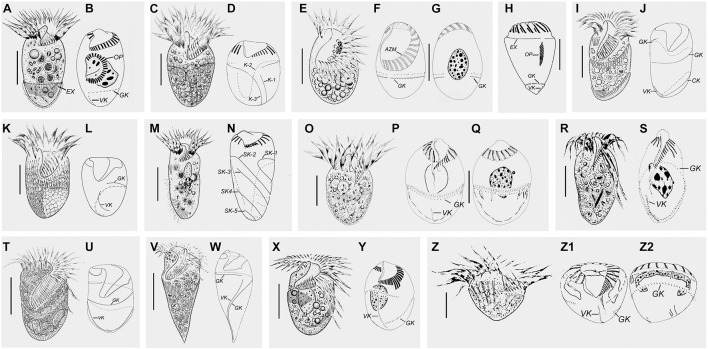
Schematic diagrams from specimens *in vivo* and after protargol staining of representative species of each strombidiid genus. **(A,B)** *Opisthostrombidium montagnesi* (after Xu et al., 2006; Agatha, 2011). **(C,D)** *Apostrombidium parakielum* (after Song W. et al., 2013). **(E–G)** *Williophrya maedai* (after Liu et al., 2011). **(H)** *Foissneridium constrictum* (after Agatha, 2011). **(I,J)** *Antestrombidium agathae* (after Liu et al., 2015b). **(K,L)** *Sinistrostrombidium cupiformum* (after Liu et al., 2015b). **(M,N)** *Varistrombidium kielum* (after Xu et al., 2011). **(O–Q)** *Strombidium sulcatum* (after Song et al., 2000). **(R,S)** *Omegastrombidium elegans* (after Song et al., 2000). **(T,U)** *Spirostrombidium faurefremieti* (after Wang et al., 2018). **(V,W)** *Parallelostrombidium jankowskii* (after Song et al., 2018). **(X,Y)** *Novistrombidium (Propecingulum) sinicum* (after Liu et al., 2009). **(Z–Z2)** *Novistrombidium (Novistrombidium) testaceum* (after Song and Bradbury, 1998). Scale bars = 20 μm **(C,E,I,K,O,X)**; 15 μm **(A,R)**; 30 μm **(M,T,Z)**; 40 μm **(V)**.

**FIGURE 3:**
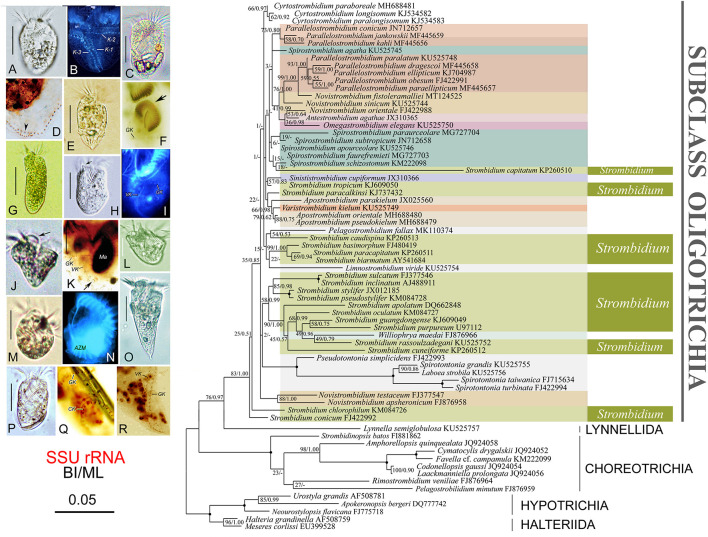
Photomicrographs of specimens *in vivo* and after protargol staining of a representative species of each strombidiid genus (left) and maximum likelihood tree inferred from SSU rRNA gene sequence data (right). **(A,B)** *Apostrombidium parakielum* (after Song W. et al., 2013). **(C,D)** *Spirostrombidium faurefremieti* (after Wang et al., 2018). **(E,F)** *Opisthostrombidium montagnesi* (after Xu et al., 2006). **(G)** *Varistrombidium kielum* (after Xu et al., 2011). **(H,I)** *Sinistrostrombidium cupiformum* (after Liu et al., 2015b). **(J,K)** *Strombidium paracapitatum* (after Song et al., 2015b). **(L)** *Novistrombidium (Novistrombidium) testaceum* (after Song W. et al., 2013). **(M,N)** *Williophyra maedai* (after Liu et al., 2011). **(O,R)** *Parallelostrombidium jankowskii* (after Song et al., 2018). **(P,Q)** *Antestrombidium agathae* (after Liu et al., 2015b). Numbers at the nodes represent support values in the following order: BI posterior probabilities and ML bootstrap values. Disagreements in topology between the BI and ML trees are indicated by a hyphen. Nodes that were maximally supported (1.00 BI; 100% ML) are represented by filled circles. Scale bar, three substitutions per 100 nucleotide positions. Species discussed in the present study are shown in bold type.

The taxonomy of the 12 known genera of Strombidiidae are summarized, including their morphological diagnostic characters, type species, remarks on the history of their establishment, and subsequent revisions (Figures 2, 3).

**Genus *Antestrombidium* (Liu et al., 2015b)**

Diagnosis: Strombidiidae with three somatic kineties, i.e., a dextrally spiraled girdle kinety, a circular kinety and a ventral kinety (Liu et al., 2015b).

**Type species:***Antestrombidium agathae* (Liu et al., 2015b)

Liu et al. (2015b) found a strombidiid with a circular kinety fragment. This is a unique character based on which the genus *Antestrombidium* was established.

**Genus *Apostrombidium* (Xu et al., 2009)**

Diagnosis: Strombidiidae with somatic kinety consisting of several fragments that extends toward the posterior end of the cell on both ventral and dorsal sides, with or without a subterminal kinety fragment. Oral primordium located between kinety fragments 1 and 2 (Song et al., 2019).

**Type species:***Apostrombidium pseudokielum* (Xu et al., 2009)

The genus *Apostrombidium* was established by Xu et al. (2009) based on the morphological characters of *Apostrombidium pseudokielum*. Later, two new species were assigned to the genus and the diagnosis for the genus was improved by supplying some new characters (Song W. et al., 2013; Song et al., 2019).

**Genus *Foissneridium* (Agatha, 2011)**

Diagnosis: Strombidiidae with oral primordium posterior to the horizontal stripe of extrusome attachment sites and anterior to the horizontal girdle kinety. Ventral kinety longitudinal (Agatha, 2011).

**Type species:***Foissneridium constrictum* (Meunier, 1910; Agatha, 2011)

Extrusome attachment sites arranged along the anterior margin of the girdle kinety are typical in Strombidiidae (Agatha, 2011). In *F. constrictum*, however, the extrusome attachment sites are pre-equatorial. Moreover, its oral primordium is posterior to the extrusome attachment sites and anterior to the girdle kinety. These are genus-level characters based on which *Foissneridium* was established.

Although the oral primordium is located anterior to their girdle kinety in both *Foissneridium* and *Opisthostrombidium*, the former differs from the latter in that its oral primordium is posterior to the extrusome attachment sites (vs. anterior to extrusome attachment sites). *Foissneridium constrictum* is the only species in the genus *Foissneridium*.

**Genus *Novistrombidium* (Song and Bradbury, 1998)**

Diagnosis: Strombidiidae with incomplete girdle kinety around the equatorial area that is conspicuously open with a large ventral gap through which ventral kinety extends (Song and Bradbury, 1998).

**Type species:***Novistrombidium testaceum* (Anigstein, 1914; Song and Bradbury, 1998)

The genus *Novistrombidium* was established by Song and Bradbury (1998). Agatha and Strüder-Kypke (2014) established two subgenera of *Novistrombidium*, mainly based on different locations of the oral primordium relative to the girdle kinety and the extrusome attachment sites. In the subgenus *Novistrombidium* (*Novistrombidium*) Song and Bradbury (1998), the oral primordium is located between a question mark-shaped field of extrusome attachment sites and the girdle kinety. In the subgenus *Novistrombidium* (*Propecingulum*) Agatha and Strüder-Kypke (2014), the oral primordium is located anterior to the stripe of extrusome attachment sites that extends alongside the girdle kinety.

Küppers et al. (2019) considered the morphological differences between these two subgenera to be sufficient for them to be elevated to genus rank. However, considering the limited numbers of known species in these two subgenera, we prefer to adopt a conservative approach pending the availability of more morphological and molecular data from more taxa.

**Genus *Omegastrombidium* (Agatha, 2004)**

Diagnosis: Girdle kinety horizontally oriented on dorsal side, extending to posterior end of body on ventral side (Agatha, 2004).

**Type species:***Omegastrombidium elegans* (Florentin, 1901; Agatha, 2004)

Florentin (1901) described this species under the name *Strombidium elegans.* Song et al. (2000) provided a redescription and revealed its ciliary pattern for the first time. Based on the girdle kinety performing a “Ω” shape, Agatha (2004) established the genus *Omegastrombidium* and designated *O. elegans* the type species.

**Genus *Opisthostrombidium* (Agatha, 2011)**

Diagnosis: Strombidiidae with oral primordium anterior to the horizontal girdle kinety and associated extrusome attachment sites (Agatha, 2011).

**Type species:***Opisthostrombidium montagnesi* (Xu et al., 2006; Agatha, 2011)

In some *Strombidium* species, the oral primordium forms anteriorly to the horizontal girdle kinety and extrusome attachment sites, thus differing from the usual arrangement in *Strombidium* species in which the oral primordium forms posteriorly to the horizontal girdle kinety and extrusome attachment sites. The genus *Opisthostrombidium* is separated from *Strombidium* based on this character.

**Genus: *Parallelostrombidium* (Agatha, 2004)**

Diagnosis: Ventral kinety follows the posterior portion of the dextrally spiraled girdle kinety; thus, both kineties have the same orientation (Agatha, 2004).

**Type species:***Parallelostrombidium rhyticollare* (Corliss and Snyder, 1986; Agatha, 2004)

Corliss and Snyder (1986) described this species under the name *Strombidium rhyticollare.* Petz et al. (1995) provided a redescription and transferred it to the genus *Spirostrombidium* based on the spiraled girdle kinety. Later, based on the similar orientation of the ventral and girdle kineties, Agatha (2004) established the genus *Parallelostrombidium* and designated *P. rhyticollare* as the type species. Agatha (2004) concluded that the ventral kinety and the posterior portion of the girdle kinety in *Parallelostrombidium* may or may not be inversely oriented.

**Genus *Sinistrostrombidium* (Liu et al., 2015b)**

Diagnosis: Strombidiidae with a ventral kinety and sinistrally spiraled girdle kinety; oral primordium develops below left end of girdle kinety.

**Type species:***Sinistrostrombidium cupiformum* (Liu et al., 2015b)

In strombidiids, the girdle kinety is generally circular or dextrally spiraled. In *S. cupiformum*, however, the girdle kinety is sinistrally spiraled, based on which the genus *Sinistrostrombidium* was established (Liu et al., 2015b).

**Genus: *Spirostrombidium* (Jankowski, 1978)**

Diagnosis: Girdle kinety dextrally spiraled, posterior portion inversely orientated and parallel to longitudinal ventral kinety (Agatha, 2004).

**Type species:***Spirostrombidium sauerbreyae* (Kahl, 1932)

The genus *Spirostrombidium* was established by Jankowski (1978) and redefined by Agatha (2004). The ciliary pattern of this genus is similar to that of *Parallelostrombidium* except that the posterior portions of the ventral kinety and girdle kinety have opposite orientations. A key to the identification of species of *Spirostrombidium* was provided by Wang et al. (2018).

**Genus *Strombidium* (Claparède and Lachmann, 1859)**

Diagnosis: Girdle kinety horizontal. Ventral kinety longitudinal, occasionally reduced, or lacking. Oral primordium develops at or below level of girdle kinety (Agatha, 2004).

**Type species:***Strombidium sulcatum* (Claparède and Lachmann, 1859)

*Strombidium* is the type and the most speciose genus in the family Strombidiidae; however, some poorly characterized nominal species have been misidentified as *Strombidium*.

**Genus *Varistrombidium* (Xu et al., 2011)**

Diagnosis: Strombidiidae with five spirally arranged somatic kineties that run obliquely across the ventral side and parallel to each other, with the longest two extending onto the dorsal side and terminating in the caudal area (Xu et al., 2011).

**Type species:***Varistrombidium kielum* (Maeda and Carey, 1985; Xu et al., 2011)

Kahl (1932) described a species collected from marine sand in Kiel Bay, Germany, and identified it as an unknown species of *Strombidium*. Maeda and Carey (1985) named the form *Strombidium kielum*. Xu et al. (2011) redescribed this species and revealed its unique ciliary pattern, based on which they established the genus *Varistrombidium.*

**Genus *Williophrya* (Liu et al., 2011)**

Diagnosis: Strombidiidae with undifferentiated adoral membranelles; somatic ciliature consisting of only a single-rowed girdle kinety that is horizontally oriented and bipartite.

**Type species:***Williophrya maedai* (Liu et al., 2011)

*Williophrya maedai* is the only species in the family Strombidiidae with undifferentiated adoral membranelles. Based on this character, Liu et al. (2011) established the genus *Williophrya*.

### Key to the Identification of Genera in the Family Strombidiidae

For the identification of each genus, detailed information on the somatic ciliary pattern, and for some genera the position of the oral primordium, is necessary.

1 Adoral zone of membranelles divided into collar and ventral
portions………………………………………………………………………………..2
1′ Adoral zone of membranelles undivided………….*Williophyra*
2 One to three somatic kineties………………………………………………….3
2′ More than three somatic kineties…………………………………….11
3 Girdle kinety horizontally oriented…………………………………………4
3′ Girdle kinety not horizontally oriented…………………………….6
4 Oral primordium located below girdle kinety…………………….
…………………………………………………………………………..*Strombidium*
4′ Oral primordium located above girdle kinety……………………5
5 Oral primordium located below extrusomes………*Foissneridium*
5′ Oral primordium above extrusomes……*Opisthostrombidium*
6 Girdle kinety “Ω” shape…………………………….*Omegastrombidium*
6′ Girdle kinety not “Ω” shape………………………………………………7
7 Girdle kinety sinistrally spiraled……………….*Sinistrostrombidium*
7′ Girdle kinety dextrally spiraled…………………………………………8
8 With circular kinety………………………………………*Antestrombidium*
8′ Without circular kinety……………………………………………………..9
9 Ventral kinety perpendicular to posterior portion of girdle
kinety………………………………………………………….*Novistrombidium*
9′ Ventral kinety not perpendicular to posterior portion of
girdle kinety…………………………………………………………………..10
10 Ventral kinety parallel with girdle kinety…………………………….
…………………………………………………………….*Parallelostrombidium*
10′ Ventral kinety inversely parallel with girdle kinety…..
………………………………………………………………*Spirostrombidium*
11 Five obliquely oriented kineties on ventral side………………..
…………………………………………………………………….*Varistrombidium*
11 ′ Two longitudinally oriented kineties on ventral side………….
……………………………………………………………….*Apostrombidium*

### Phylogeny of Strombidiid Genera Based on SSU rRNA Gene Sequences

The tree topologies from the BI and ML analyses are similar; therefore, only the ML tree is presented with support values from both methods at the branch nodes (Figure 3). DNA sequences have been reported for 10 of the 12 genera in the family Strombidiidae, the exceptions being *Foissneridium* and *Opisthostrombidium*.

Each of the genera *Strombidium* and *Spirostrombidium* is polyphyletic, which is consistent with previous studies (Wang et al., 2018; Song et al., 2020). To date, there is no agreed interpretation of their polyphyly. Species of *Strombidium* fall into several assemblages, some with morphological support: (1) *Strombidium conicum* and *S. chlorophyllum* are basal within the Oligotrichia, and their close relationship corresponds to their morphological similarity in that both species share a special kind of hemitheca; (2) *Strombidium basimorphum*, *S. paracapitatum*, and *S. biarmatum* form a well-supported clade which corresponds with their morphological similarity in that each has two types of extrusome whereas all other strombidiids have only one; (3) *Strombidium apolatum*, *S. rassoulzadegani*, *S. oculatum*, *S. purpureum*, *S. guangdongense*, and *S. cuneiforme* fall into a well-supported clade that also includes *Williophrya maedai* (present work; Liu et al., 2011, 2013, 2015a, 2016; Song W. et al., 2013; Song et al., 2015a,b, 2018, 2019; Wang et al., 2018). This has been referred to as the “eyespot clade” since all species within it possess a pigment spot, although *S. purpureum* lacks detailed *in vivo* information (Gao et al., 2016; Liu et al., 2016). *Spirostrombidium* is polyphyletic, although the positions of some *Spirostrombidium* species are not stable and have only low statistical support, which is consistent with previous studies (Wang et al., 2018; Song et al., 2020).

The genus *Parallelostrombidium* forms two clusters that correspond to differences in cell shape and somatic ciliary pattern, which is consistent with previous phylogenetic studies (Liu et al., 2013, 2015a; Song et al., 2018). One cluster comprises *Parallelostrombidium conicum*, *P. jankowskii*, and *P. kahli*, each of which has an obconical cell shape with a pointed posterior end and their ventral kinety parallel to the girdle kinety except the anteriormost portion. The other cluster comprises five species that share a dorsoventrally flattened cell shape with a rounded posterior end, and only the posterior portion of the ventral kinety is parallel to the girdle kinety.

Species of *Novistrombidium* are divided into two distantly related assemblages, which corresponds with the separation of this genus into two subgenera and may support their raising to genus rank (Agatha and Strüder-Kypke, 2014; Küppers et al., 2019). The subgenus *Novistrombidium* (*Novistrombidium*) is monophyletic whereas the subgenus *Novistrombidium* (*Propecingulum*) is not monophyletic as each of its species clusters with other genera.

The three *Apostrombidium* species and *V. kielum* form a clade (Figure 3), and previous studies have consistently recovered a close relationship between *Apostrombidium* and *Varistrombidium* (Liu et al., 2013, 2016; Song W. et al., 2013; Song et al., 2015a, 2019; Tsai et al., 2015). Morphological data support this finding since both genera have a dorsal split of the girdle kinety and long cilia on the dorsal side (Gao et al., 2016).

In the BI tree (not shown), *Antestrombidium agathae* clusters with *Omegastrombidium elegans* and then clusters with *Novistrombidium orientale*, whereas in the ML tree *A. agathae* clusters with *N. orientale*, which together cluster with *Omegastrombidium elegans*. In several previous studies, *A. agathae* clusters with *O. elegans* (Liu et al., 2015b, 2016; Gao et al., 2016; Song et al., 2019). This finding supports the close evolutionary relationship between *Antestrombidium* and *Omegastrombidium* hypothesized in Liu et al. (2015b) and corresponds with the similarity of their morphology. For example, the circular kinety of *Antestrombidium* appears to be homologous to the Ω-shaped girdle kinety in *Omegastrombidium*.

*Sinistrostrombidium cupiformum* clusters with *Strombidium tropicum*, which is consistent with previous studies (Liu et al., 2015b; Wang et al., 2018; Song et al., 2019). It is noteworthy that the girdle kinety of *Strombidium tropicum* is slightly spiraled, i.e., the left end of the girdle kinety is positioned higher than the right one, and may represent a ciliary pattern from which *Sinistrostrombidium* originated (Liu et al., 2015a,b). Nevertheless, the evolutionary relationship between these two species requires further investigation since previous studies have reported that *Sinistrostrombidium cupiformum* forms an isolated basal branch in some phylogenetic tress (Gao et al., 2016; Liu et al., 2016).

### Geographic Distribution of the Strombidiids in China

**FIGURE 4:**
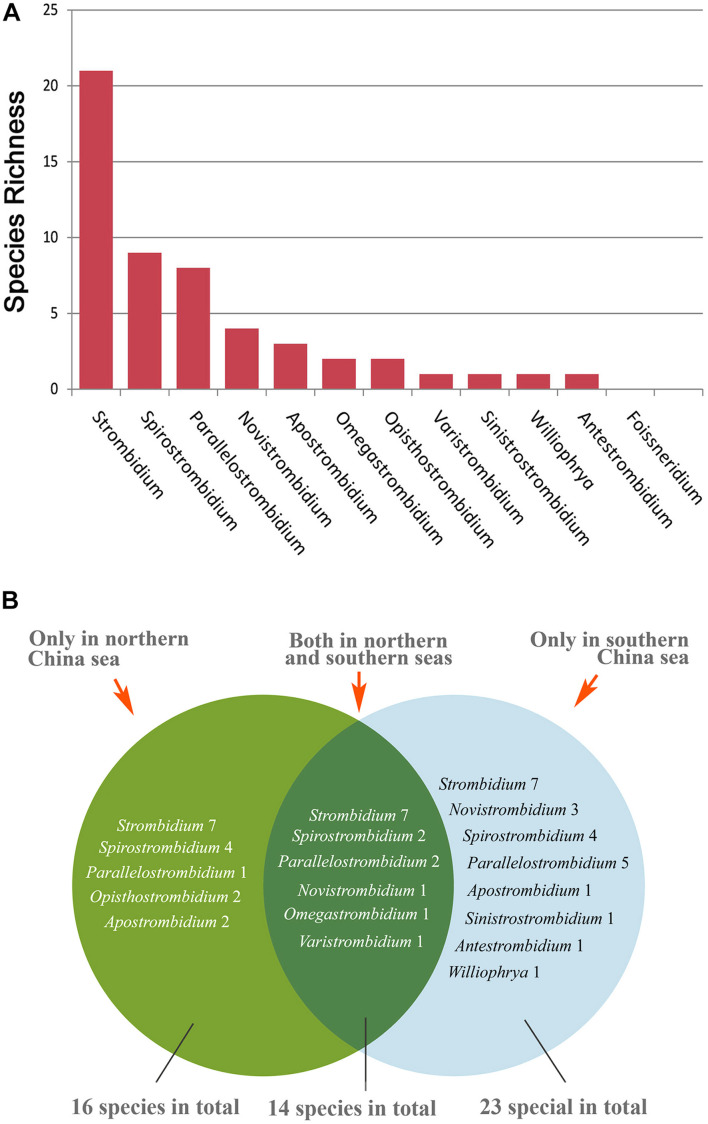
Diversity of strombidiids collected from China **(A)** Species richness of each genus. **(B)** Comparison of species composition between coastal waters of northern and southern China.

Eleven of the 12 genera of the family Strombidiidae have been found in Chinese coastal waters (Figures 1, 4), the exception being *Foissneridium* which has so far only been isolated from the Barents Sea (Meunier, 1910). In terms of species numbers, the genus *Strombidium* is best represented with 21 species (Figure 4A). This is consistent with findings in other geographic regions such as the northwest and south Atlantic Ocean, the Baltic Sea, and the Mediterranean Sea, where *Strombidium* is also the strombidiid genus represented by the largest number of species (Dolan and Marrasé, 1995; Santoferrara and Alder, 2009; Agatha, 2011). Ranking second and third are *Spirostrombidium* (nine species) and *Parallelostrombidium* (eight species), respectively (Figure 4A). For *Varistrombidium*, *Williophrya*, *Sinistrostrombidium*, and *Antestrombidium*, only one species each has been recorded in Chinese coastal waters. Regarding the distribution of these genera, *Strombidium* has a most extensive range with occurrences at 15 sites, followed by *Parallelostrombidium* (10 sites) and *Spirostrombidium* (nine sites) (Figure 1B). The comparison of diversity in these sites showed that the highest species richness (14 species) occurred at site k and the lowest was at five sites (h, i, l, n, p) with only one species at each (Figure 1C). At the genus level, sites a, k, and q were the most genus-rich with six genera at each whereas sites d, h, i, l, n, and p each had only one genus (Figure 1B).

Occurrences of strombidiids in northern and southern China were compared (Figure 4B). Representative of seven genera, i.e., *Strombidium*, *Spirostrombidium*, *Parallelostrombidium*, *Novistrombidium*, *Omegastrombidium*, *Apostrombidium*, and *Varistrombidium*, were found both in northern and southern China. *Opisthostrombidium* has only been reported from northern Chinese coastal waters, while *Williophrya*, *Sinistrostrombidium*, and *Antestrombidium* have only been reported from southern Chinese coastal waters. The total number of strombidiids is higher in southern China where 37 species representing 10 genera were collected compared to 30 species and eight genera in northern China. There are two possible reasons for this: (1) the sampling area was wider and the number of sampling sites was higher in southern than in northern China; (2) the environment features of some southern coastal habitats are more suitable for ciliates than those in the north. For example, mangrove wetlands, which are nutrient-rich and could provide a greater quantity and diversity of food resources for ciliates, are only located in southern China.

### Global Distribution of the Strombidiids Based on Molecular Information

Most Strombidiidae species for which gene sequence data (mostly SSU rRNA but also ITS and LSU rRNA sequences of some taxa) are available in the GenBank database are from specimens collected from coastal water habitats, including sediments, lagoons, and bays, which are much easier to sample than oceanic habitats. To infer the possible worldwide distribution of the reported species within Strombidiidae, BLAST comparisons against the GenBank Nucleotide collection database were run with SSU rRNA gene sequences of known species. The Sequence Read Archive (SRA) contains only short sequences from high throughput sequencing and thus was not used in the present study.

**FIGURE 5:**
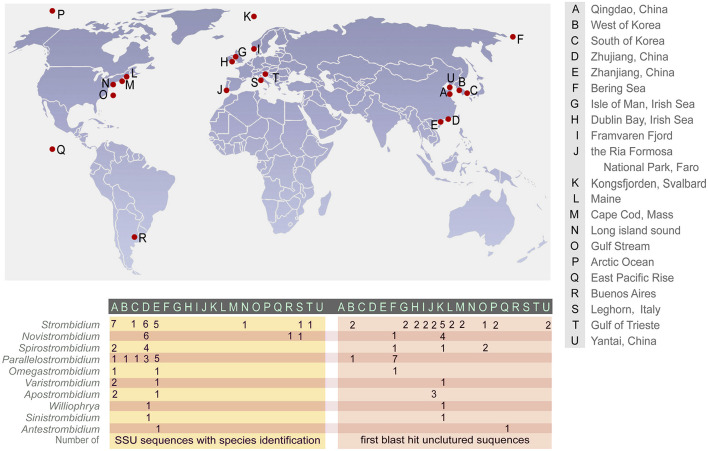
Global geographic distribution of the 12 genera of Strombidiidae based on SSU sequences.

The nearest environmental neighbor (NEN) for each species, i.e., the environmental sequence with the highest sequence similarity, was obtained as the first BLAST hit. The NENs of strombidiids were from various habitats including mangroves, coastal waters, estuaries, fjords, solar saltern ponds, open ocean waters, and oxygen-depleted marine environments (Figure 5 and Table 1). *Parallelostrombidium conicum* was identical to its NEN (100% similarity), which was reported from the Bering Sea (KC771186). Five *Strombidium* species, four *Novistrombidium* species, three *Apostrombidium* species, three *Spirostrombidium* species, *Sinistrostrombidium cupiformum*, and *Varistrombidium kielum* each had the same NEN, i.e., EU371386, which was collected from Kongsfjorden, Svalbard (Table 1). Similarly, seven out of eight *Parallelostrombidium* species, *Omegastrombidium elegans*, *Spirostrombidium schizostomum*, and *Novistrombidium fistoleramalliei* also had the same NEN, i.e., KC771186, which was collected from the Bering Sea. The NENs of *Strombidium rassoulzadegani* and *S. intermedium*, and of *S. basimorphum* and *S. paracapitatum*, were collected from an oxygen-depleted salt marsh in Massachusetts, United States (AY180033) and the anoxic Framvaren fjord in Norway (EF527106), respectively.

**TABLE 1:** Locations of SSU rRNA gene sequences and their nearest environmental neighbor (NEN).

**Species name**	**Location**	**GenBank No.**	**Similarity (NEN)**	**GenBank No. (NEN)**	**Location (NEN)**
*Antestrombidium agathae*	E	JX310365	98.03	KJ757606	Q
*Apostrombidium pseudokielum*	A	MH688479	99.34	EU371386	K
*Apostrombidium parakielum*	A	JX025560	97.92	EU371386	K
*Apostrombidium orientale*	E	MH688480	99.24	EU371386	K
*Novistrombidium apsheronicum*	D	FJ876958	95.89	EU371386	K
*Novistrombidium orientale*	D	FJ422988	97.97	EU371386	K
*Novistrombidium sinicum*	D	FJ422989	98.03	EU371386	K
*Novistrombidium sinicum*	D	KU525744	98.01	EU371386	K
*Novistrombidium sinicum*	D	FJ422990	98.03	EU371386	K
*Novistrombidium testaceum*	D	FJ377547	96.51	EU371386	K
*Novistrombidium testaceum*	S	AJ488910	96.95	EU371386	K
*Novistrombidium fistoleramalliei*	R	MT124525	97.61	KC771186	F
*Omegastrombidium elegans*	A	EF486862	96.71	KC771186	F
*Omegastrombidium elegans*	E	KU525750	97	KC771186	F
*Parallelostrombidium conicum*	E	JN712657	100	KC771186	F
*Parallelostrombidium dragescoi*	E	MF445658	96.82	KC771186	F
*Parallelostrombidium ellipticum*	D	KJ704987	96.84	KC771186	F
*Parallelostrombidium jankowskii*	E	MF445659	99	KC771186	F
*Parallelostrombidium kahli*	A	MF445656	99	KC771186	F
*Parallelostrombidium obesum*	E	KU525733	90.3	FJ032675	B
*Parallelostrombidium obesum*	D	FJ422991	97.24	KC771186	F
*Parallelostrombidium paraellipticum*	D	MF445657	96.74	KC771186	F
*Parallelostrombidium paralatum*	E	KU525748	97.8	KC771186	F
*Parallelostrombidium paralatum*	B	KF800042	96.67	KC771186	F
*Parallelostrombidium paralatum*	C	HM140404	96.84	KC771186	F
*Sinistrostrombidium cupiformum*	D	JX310366	98.84	EU371386	K
*Spirostrombidium subtropicum*	E	JN712658	98.72	EU371386	K
*Spirostrombidium apourceolare*	E	KU525746	99.52	KJ760066	O
*Spirostrombidium agathae*	A	KU525745	98.63	KJ760066	O
*Spirostrombidium schizostomum*	D	KM222098	96.44	KC771186	F
*Spirostrombidium faurefremieti*	A	MG727703	99.4	EU371386	K
*Spirostrombidium paraurceolare*	E	MG727704	96.44	EU371386	K
*Strombidium apolatum*	A	DQ662848	99.71	GU206560	G, H, L
*Strombidium biarmatum*	T	AY541684	99.78	KJ761932	P
*Strombidium basimorphum*	D	FJ480419	98.65	EF527106	I
*Strombidium rassoulzadegani*	N	AY257125	99.94	AY180033	M
*Strombidium rassoulzadegani*	A	KU525752	98.55	AY180033	M
*Strombidium capitatum*	A	KP260510	98.17	JX178772	U
*Strombidium caudispina*	E	KP260513	99.87	KJ759113	P
*Strombidium chlorophilum*	A	KM084726	96.96	KJ760066	O
*Strombidium conicum*	D	FJ422992	97.81	EU371386	K
*Strombidium cuneiforme*	E	KP260512	96.95	GU206562	G
*Strombidium guangdongense*	E	KJ609049	99.2	KR028987	J
*Strombidium intermedium*	A	KX131153	95.61	AY180033	M
*Strombidium inclinatum*	S	AJ488911	96.38	EU371386	K
*Strombidium oculatum*	D	KM084727	97.67	GU206561	H
*Strombidium paracapitatum*	D	KP260511	99.04	EF527106	I
*Strombidium paracalkinsi*	C	KJ737432	99.04	EU371386	K
*Strombidium pseudostylifer*	E	KM084728	98.92	FJ543106	B
*Strombidium stylifer*	D	JX012185	99.49	FJ543106	B
*Strombidium stylifer*	A	DQ631805	99.55	FJ543106	B
*Strombidium sulcatum*	A	FJ377546	96.23	EU371386	K
*Strombidium sulcatum*	A	DQ777745	96.45	EU371386	K
*Strombidium triquetrum*	E	KJ609052	99.71	JX178772	U
*Strombidium tropicum*	D	KJ609050	99.54	EU371386	K
*Varistrombidium kielum*	E	KJ609051	97.8	EU371386	K
*Varistrombidium kielum*	A	KU525749	98.97	EU371386	K
*Varistrombidium kielum*	A	DQ811090	98.31	EU371386	K
*Williophrya maedai*	D	FJ876966	95.32	KR028987	J

*For explanation of locations, see Figure 5.*

For environmental sequencing studies, delineating operational taxonomic units (OTUs) is a key step in the analysis and can significantly affect the results (Nebel et al., 2011). A 5% cutoff (95% sequence similarity) was recommended for microbial eukaryotes by Caron et al. (2009). For ciliates, a 1%–3% cutoff value (i.e., 97–99% sequence similarity) for the SSU rRNA gene is often used (Stoeck et al., 2006; Doherty et al., 2007, 2010; Sun et al., 2017, 2019), although a finer (<1%) cutoff might be needed for some groups (Xu et al., 2013). In the present analysis, the average similarity of the 49 species/populations of strombidiids was 97.9% with their NENs, ranging from 90.3 to 100%. Among these, 17 species/populations had a similarity with their NEN of >99%, 15 had a similarity of 97–99%, 16 had a similarity of 95–97%, and one had a similarity of 90.3%. If a 97% similarity cutoff is used to distinguish different species, 65% of the 49 species/populations are conspecific with their NEN. Even if a much stricter cutoff is used (1%), 35% of the species/populations are conspecific with their NEN. These findings suggest that the species isolated from Chinese coastal waters were likely to be globally distributed based on the 5% cutoff suggested by Caron et al. (2009) with 48 out of 49 species being conspecific with their NEN. Furthermore, it is noteworthy that some species from different genera can have the same NEN, which may indicate that the environmental sampling and sequencing efforts are far from saturation. Increased collection of ciliate environmental sequences from different marine environments, especially those that are difficult to sample, will help improve knowledge and understanding of the biogeographical distribution patterns of marine strombidiids. Also, the enrichment of the full-length or near full-length SSU rRNA gene sequences from various oceanic environments paired with detailed morphological observations will serve to improve the database, thereby contributing to the identification of environmental sequences.

## Data Availability Statement

The original contributions presented in the study are included in the article/supplementary material, further inquiries can be directed to the corresponding author/s.

## Author Contributions

LL, WeiS, and MS conceived the research. WenS, DX, and XC conducted the analysis and drafted the manuscript. AW critically reviewed the findings and improved the manuscript. All authors contributed to the article and approved the submitted version.

## Conflict of Interest

The authors declare that the research was conducted in the absence of any commercial or financial relationships that could be construed as a potential conflict of interest. The handling editor declared a past co-authorship with one of the authors LL.

## Publisher’s Note

All claims expressed in this article are solely those of the authors and do not necessarily represent those of their affiliated organizations, or those of the publisher, the editors and the reviewers. Any product that may be evaluated in this article, or claim that may be made by its manufacturer, is not guaranteed or endorsed by the publisher.
